# Bifurcation in Blood Oscillatory Rhythms for Patients with Ischemic Stroke: A Small Scale Clinical Trial using Laser Doppler Flowmetry and Computational Modeling of Vasomotion

**DOI:** 10.3389/fphys.2017.00160

**Published:** 2017-03-23

**Authors:** Alexey Goltsov, Anastasia V. Anisimova, Maria Zakharkina, Alexander I. Krupatkin, Viktor V. Sidorov, Sergei G. Sokolovski, Edik Rafailov

**Affiliations:** ^1^Division of Science, School of Science, Engineering and Technology, Abertay UniversityDundee, UK; ^2^Department of Neurology, Neurosurgery and Medical Genetics, Pirogov Russian National Research Medical University, First City HospitalMoscow, Russia; ^3^Department of Functional Diagnostics, Priorov's Central Institute of Traumatology and OrthopedicsMoscow, Russia; ^4^SPE “LAZMA” Ltd.Moscow, Russia; ^5^Optoelectronics and Biomedical Photonics Group, Photonics and Nanoscience Group, Aston Institute of Photonic Technologies, Aston UniversityBirmingham, UK

**Keywords:** laser Doppler flowmetry, acute ischemic stroke, cerebrovascular haemodynamics, blood flow oscillation, vasomotion modeling

## Abstract

We describe application of spectral analysis of laser Doppler flowmetry (LDF) signals to investigation of cerebrovascular haemodynamics in patients with post-acute ischemic stroke (AIS) and cerebrovascular insufficiency. LDF was performed from 3 to 7 days after the onset of AIS on forehead in the right and left supraorbital regions in patients. Analysis of LDF signals showed that perfusion in the microvasculature in AIS patients was lower than that in patients with cerebrovascular insufficiency. As a result of wavelet analysis of the LDF signals we obtained activation of the vasomotion in the frequency range of myogenic oscillation of 0.1 Hz and predominantly nutritive regime microcirculation after systemic thrombolytic therapy of the AIS patients. In case of significant stroke size, myogenic activity, and nutritive pattern microhaemodynamics were reduced, in some cases non-nutritive pattern and/or venular stasis was revealed. Wavelet analysis of the LDF signals also showed asymmetry in wavelet spectra of the LDF signals obtained in stroke-affected and unaffected hemispheres in the AIS patients. A mechanism underlying the observed asymmetry was analyzed by computational modeling of vasomotion developed in Arciero and Secomb (2012). We applied this model to describe relaxation oscillation of arteriole diameter which is forced by myogenic oscillation induced by synchronous calcium oscillation in vascular smooth muscle cells. Calculation showed that vasomotion frequency spectrum at the low-frequency range (0.01 Hz) is reciprocally modulated by myogenic oscillation (0.1 Hz) that correlates with experimental observation of inter-hemispheric variation in the LDF spectrum.

## Introduction

Laser near-infrared spectroscopy (NIRS) and laser Doppler flowmetry (LDF) techniques along with spectral analysis of the optical signals become powerful method in clinical diagnostics of cerebrovascular diseases (Smith, 2011; Reinhard et al., 2003; Semyachkina-Glushkovskaya et al., 2016). High time resolution of NIRS and LDF methods allows measurement not only mean tissue oxygen saturation and blood flow (perfusion) but small hemocirculation fluctuations in cerebral oxygenation haemodynamic and metabolic changes (Mitra et al., 2016). These spectroscopic data were reported to bear valuable information on control of cerebral circulation in healthy and pathology (Schytz et al., 2010; Reinhard et al., 2014). One of directions in the analysis of medical optical spectra aims at the investigation of cerebrovascular autoregulation (CA) and its impairment as a result of cerebrovascular diseases. Using of NIRS and LDF methods in clinics allows non-invasive measurement of CA instead of invasive common techniques (Fantini et al., 2016) and the development of the multifunctional, inexpensive portable technologies enabling clinicians to easily perform continuous monitoring of patients during diagnosis, treatment, and posttreatment periods (Rafailov et al., 2015).

NIRS and LDF spectra contain information on temporal variation of the mean arteria blood pressure (MABP), cerebral blood flow (CBF), and oxygenation. They include both passive components such as heart and breathing pulses and the active ones such as spontaneous oscillation in low-frequency oscillation range from 0.05 to 0.15 Hz (LFO) and very low low-frequency oscillation ranged up to 0.05 Hz (VLFO) bands (Schytz et al., 2010). Specific frequency bands in optical spectra were reported to link to CA which is a critical mechanism to maintain stable blood flow in an ischemic liaison area. A change in CA is considered as an important factor in the diagnosis and treatment of cerebrovascular diseases (Reinhard et al., 2012; Liu et al., 2015). The most frequently used parameters for analysis of CA are phase shift (PS) in the frequency domains of LFO or VLFO and correlation coefficients in the time domains between MABP/CBF and oxygenated (oxyHb)/deoxygenated (deoxyHb) hemoglobin (Schytz et al., 2010; Reinhard et al., 2014). Clinical investigation of ischemic patients showed an increase in the correlation coefficients obtained for ischemic affected and non-affected hemispheres that indicates weakening CA (Reinhard et al., 2012).

To extract information on cerebrovascular dynamics and CA abnormalities from NIRS and LDF signals, different techniques of spectral analysis are used, among which are Fourier transformation (Rossi et al., 2007; Vermeij et al., 2014), wavelet analysis (Stefanovska, 1999; Addison, 2015), signal correlation analysis (Toronov et al., 2013), and signal linear and nonlinear decomposition methods (Iatsenko et al., 2015; Caicedo et al., 2016). For further development and translation of the spectral analysis of NIRS and LDF signals as a clinical method, it will need detailed investigation of the molecular and physiological mechanisms behind LFO and VLFO in healthy and pathology (Schytz et al., 2010). Although spectral methods are intensively used in investigation of CA in pathological conditions, the link of these active oscillations with cerebrovascular diseases is not sufficiently explored. At present, investigation of a link between LFO amplitude and cerebrovascular diseases showed a significant response in spectral characteristics to such pathological conditions as carotid stenosis (Hu et al., 1999; Schytz et al., 2010), acute ischemic stroke (AIS) (Phillip and Schytz, 2014), traumatic brain injury (Chernomordik et al., 2016), and cerebrovascular occlusive disease (Phillip et al., 2013).

Intensive investigation of the spontaneous oscillation in microvascular flow, referred as vasomotion (oscillation of vascular diameter), revealed that its spectral characteristics are intrinsic hallmarks of microvascular regulation of blood flow in different tissues and organs (Kvandal et al., 2003). As a result of the spectral analysis of NIRS and LDF signals, several inherent frequency regions were identified and attributed to different physiological mechanisms of vascular control: (I) 0.6–2 Hz related to cardiac activity, (II) 0.145–0.6 Hz corresponds to respiratory activity, (III) 0.052–0.145 Hz linked to microvessel smooth muscle cell activity, (IV) 0.021–0.052 Hz connected to microvessel innervation, and region (V) 0.005 Hz–0.0095 Hz associated to endothelia activity (Stefanovska, 1999; Rossi et al., 2008). Experimental investigation of vasomotion are accompanied by detailed computational modeling of its molecular mechanisms (Ursino et al., 1996; Koenigsberger et al., 2008; Arciero and Secomb, 2012). Results of the experimental and theoretical investigation are assumed that different mechanisms modulate vascular smooth muscle (VSM) activity in the corresponding frequency regions of the vasomotion spectrum. Detailed investigation of the vasomotion spectrum showed a significant response of the spectral components to patho-physiological conditions such as malignant melanoma (Lancaster et al., 2015), surgical trauma and anesthesia (Schmidt-Lucke et al., 2002), arterial hypertension, skin post-ischaemic reactive hyperaemia (Rossi et al., 2007, 2008), and experimental intervention such as the endothelium-dependant vasodilators (Kvandal et al., 2003). Clinical studies demonstrated that the spectral analysis of LDF signals in the specific frequency regions is a promising method of monitoring microvessel regulation in patients with cerebrovascular diseases (Rossi et al., 2008; Krupatkin and Sidorov, 2016).

In this work we investigated a feasibility of spectral analysis of LDF signals to evaluate a function status of cerebral microcirculation system in patients with acute and chronic disorder of cerebral circulation (Krupatkin, 2005). The developed method is based on the wavelet spectral analysis of an alteration in spectral components within different frequency regions of LDF spectrum discussed above. We also applied a computational model of vasomotion developed in Ursino et al. (1996) and Arciero and Secomb (2012) to analyse possible mechanisms underlying a change in LDF spectra observed in AIS patients.

## Methods

Patient investigation was carried out in the Regional Vascular Centre of the Pirogov Russian National Research Medical University (RNRMU), Moscow, Russia. Clinical trial included 19 patients, 10 man and 9 women, aged from 27 to 76 years with the ischemic monohemispheric stroke and 12 of them were treated with thrombolytic therapy. The trial was carried out in accordance with the Declaration of Helsinki principles and approved by the Ethics Committee of the Pirogov Russian National Research Medical University. All patients participated in this trial have given the full consent on measurements in written and been informed of their right to discontinue participation at any time.

An initial average National Institute of Health Stroke Scale (NIHSS) score of the patients was 10.2 ± 4.7. Sixteen patients were classified with large-vessel disease and 13 patients with concurrent or undetermined causes according to TOAST criteria (Adams et al., 1993). Exclusion criteria in this trial were hemorrhagic stroke, cancer, infectious diseases including infections of the nervous system, mental disease, autoimmune disease, drug addiction, chronic alcoholism. Patients with AIS were evaluated by course of neurological, psychoemotional state, cognitive function, and laboratory results. The main characteristics of the patients are shown in Tables 1, 2. As a reference cohort, 22 patients with chronic cerebrovascular encephalopathy were evaluated. Nineteenth percentage of patients in this group had a combination of arterial hypertension with atherosclerosis and in the remaining group of patients, arterial hypertension was more likely to be a leading factor because it was developed in young age.

**Table 1:** **Characteristics of the patients with hemisphere stroke (group ***N*** = 19)**.

**Sex**	**Age**			**Localisation**		**Etiological factor**		**Type of insult**	
	**<45**	**45–60**	**>60**	**Right**	**Left**	**AH**	**AS+AH**	**CE**	**AT**
Male	1	5	4	6	4	2	6	7	3
Female	2	3	4	4	5	7	7	6	3
Total	3	8	8	10	9	9	13	13	6

*AH, arterial hypertension; AS, atherosclerosis; CE, cerebral embolism; AT, arterial thrombosis*.

**Table 2:** **Severity of patient status with hemisphere insult according to NIHSS score**.

**NIHSS score**	**Number of patients**
<7	7
8–13	9
>14	3
Total number	19

LDF investigation was performed from the third to seventh day after the onset of ischemic stroke. Measurements were carried out on forehead in the left and right supraorbital regions over brow skin areas corresponding to a branch of the internal carotid (ophthalmic) arteries, which supplies blood to the supraorbital artery from internal carotid artery. LDF measurement was carried out using multifunctional laser device LAKK-M (SPE LAZMA Ltd., http://www.lazma.ru; Dunaev et al., 2014; Rafailov et al., 2015). LDF signals were measured up to 300 s with time step Δt = 0.05 s. Cerebral microcirculation parameter was obtained as a value of the LDF signals in arbitrary perfusion units (pu). Then LDF signals were analyzed using wavelet transformation to investigate spectral components in the specific frequency regions of cerebral microcirculation oscillations (Kvernmo et al., 1998). Statistical analysis included calculation of the means and standard deviations of the values of arbitrary perfusion and amplitudes of the main peaks in the wavelet spectra.

### Analysis of wavelet spectrum of LDF signal

Spectral analysis of the LDF signal was carried out by the wavelet method with further determination of leading components in the specific frequency ranges of the spectrum. Following the method (Kvernmo et al., 1998), continuous wavelet transform of the LDF signal *S*(*t*) was calculated as

(1) $$W\left(s,t_{0}\right)=\frac{1}{\sqrt{s}}\underset{-\infty }{\int^{\infty }}S\left(t\right)\psi \left(\frac{t-t_{0}}{s}\right)dt$$

where $\psi \left(\frac{t-t_{0}}{s}\right)$ is the mother wavelet function which was chosen in the Morlet wavelet form

(2) $$\psi \left(v\right)=\pi^{-1/4}\exp\left(-i2\pi f_{0}v\right)\text{exp}(v^{2}/f_{1})$$

where *f*_0_ and *f*_1_ are dimensionless parameters, defining the wavelet center frequency and wavelet bandwidth respectively and $v=\frac{t-t_{0}}{s}$ is a dimensionless variable with the wavelet scales *s* and *t*_0_. Finally, we calculated the wavelet power spectrum |*W*(*s*, *t*_0_)|^2^.

### Fourier analysis of LDF spectrum

Spectral characteristics of the LDF signals were also analyzed using spectral density of auto-correlation function of the LDF signals which was calculated as

(3) $$R\left(\tau \right)=\frac{\sum \left(S(t)-\bar{S})(S(t-\tau )-\bar{S}\right)}{R\left(0\right)},$$

where $\bar{S}$ is the average LDF signal over the measurement time *T*. The power spectral density (PSD) was calculated as discrete Fourier transformation of the auto-correlation function of the LDF signal *R*(τ):

(4) $$R(f_{k})=\sum R(\tau )e^{-i2\pi k\frac{n}{N}},$$

which is defined for discrete frequencies $f_{k}=\frac{k}{T},\;$ where *k* = *N* − 1. PSD was calculated as

(5) $$P\left(f_{k}\right)=\frac{1}{N^{2}}{\left|R\left(f_{k}\right)\right|}^{2}.$$

### Computational modeling of vasomotion

To analyse the mechanism underlying the observed changes in the LDF spectra measured in AIS patients, we applied a nonlinear model of spontaneous (vasomotion) oscillation of microvessels which was developed in a series of theoretical papers (Ursino et al., 1996; Koenigsberger et al., 2008; Arciero and Secomb, 2012). The developed phenomenological models describe oscillation of arteriole diameter and blood flow under the vessel wall tension and share stresses. In these models, vasomotion involves oscillating contraction of the vascular smooth muscles (VSMs) in the arteriole wall which results from interaction between the mechanical tension of the vessel wall and the dynamics of tone generation in VSMs. In this work, we used a version of the vasomotion model developed in Arciero and Secomb (2012) and generalized it to consider myogenic oscillations of the arteriole wall which is suggested to be induced by synchronous calcium oscillation of VSMs. A kinetic model of this molecular mechanism of myogenic oscillations was developed in Koenigsberger et al. (2005). In our model, myogenic oscillations were phenomenologically considered as forcing tension induced by VSMs applied to the microvessel wall.

The model of spontaneous vasomotion of arteriole diameter (Arciero and Secomb, 2012) includes two ordinary differential equations (ODEs) which describe: (1) a change in diameter *D* of an arteriole and (2) the response of vascular smooth muscles *A* (vascular tone) to the tension in the wall *T* and wall share stress τ:

(6) $$\{\begin{array}{l} \frac{dD}{dt}=\frac{1}{\tau_{d}}\frac{D_{0}}{T_{0}}\left(T(D)-T_{tot}(D)\right)\\ \frac{dA}{dt}=\frac{1}{\tau_{a}}\left(A(D)-A_{tot}(D)\right) \end{array}$$

Here *T*(*D*) = *PD*/2 is the wall tension due to intravascular pressure *P* according to Laplace law. *T*_*tot*_(*D*) is the tension generated by VSMs in the vessel wall which is defined by its passive *T*_*pass*_(*D*) and active *T*_*act*_(*D*) components (Arciero and Secomb, 2012):

(7) $$T_{tot}\left(D\right)=T_{pass}\left(D\right)+\;A\left(D\right)T_{act}\left(D\right),$$

where

(8) $$T_{pass}\left(D\right)=C_{pass}exp\left(C_{pass}^{\prime }\left(\frac{D}{D_{0}}-1\right)\right),$$

(9) $$T_{act}\left(D\right)=C_{act}exp\left(-\frac{D/D_{0}-C_{act}^{\prime }}{C_{act}^{\prime \prime }}\right).$$

Activation function *A*(*D*) varies between 0 and 1 and takes a steady-state value *A*_*tot*_ which is defined as a sigmoidal function of a stimulus *S*:

(10) $$A_{tot}\left(S\right)={\left(1+e^{-S}\right)}^{-1},$$

where

(11) $$S=C_{myo}T-C_{shear}\tau +C_{tone}^{\prime }.$$

The first term in function *S* corresponds to the myogenic response to the wall tension. The second term describes the shear-dependence response and *C*_*tone*_ is a constant. Tension of VSMs *T* is produced by intravascular pressure and the wall share stress τ results from blood flow in small vessels. Constants *D*_0_ and *T*_0_ are normalized values of diameter and tension which were taken equal to passive reference vessel diameter and tension value *T* at *D* = *D*_0_ (Table 3). The time scales τ_*d*_ and τ_*a*_ were selected to reproduce satisfactory low-frequency spontaneous oscillations in the range of 0.01 Hz observed in the LDF spectrum. The obtained values τ_*d*_ = 5 s and τ_*a*_ = 60 s are close to those given in Arciero and Secomb (2012).

**Table 3:** **Parameters of the vasomotion model taken from Arciero and Secomb (2012)**.

**Parameters**	**Parameter values**	**Parameters**	**Parameter values**
*P*	90 mmHg (120 kdyn/cm^2^)	${C^{\prime \prime }}_{act}$	0.3
*C_*pass*_*	1.04 kdyn/cm	*C_*myo*_*	0.01 cm/dyn
${C^{\prime }}_{act}$	8.3	*C_*shear*_*	0.03 cm^2^/dyn
*D_*0*_*	156 μm	τ	8 dyn/ cm^2^
*C*_*act*_	1.6 kdyn/cm	${C^{\prime }}_{tone}$	−2.2; −5.7 in this model
${C^{\prime }}_{act}$	0.68		

We supposed that myogenic oscillations of small arteries and arterioles containing VSMs are generated by synchronous calcium oscillations in smooth muscle cells (SMCs) which force oscillations of vessel diameter (Koenigsberger et al., 2008). To describe these myogenic oscillations of the vascular diameter, we introduced additional tension *T*_*m*_ into Equation (8) in the form of forcing oscillatory function of amplitude *A*_*m*_ and frequency *f*_*m*_:

(12) $$T_{m}=A_{m}sin\left(2\pi f_{m}t\right).$$

Frequency of the myogenic oscillation in the model was taken *f*_*m*_ = 0.15 Hz that corresponds to the frequency region where myogenic oscillation was observed (Stefanovska, 1999).

In the model, we suggested that oscillations of the LDF signals result from the oscillations of the platelet velocity induced by active oscillations of the arteriole radius. Arteriole radius oscillations cause fluctuations in the hydraulic viscous resistance of an arteriole which are inversely proportional to the fourth power of the arteriole radius *r* according to Hagen-Poiseuille law, *R* = 8η*L*/*r*^4^, where *L* is the length of a vessel segment and η is blood viscosity. The oscillations of the hydraulic viscous resistance cause oscillations in blood flow in the vessel segment which is defined by Poiseuille law,

(13) $$Q=\frac{\pi \Delta P}{R},$$

where Δ*P* is the pressure difference across the arteriole segment. Thus, oscillation of the LDF signals, which is proportional to *Q*, is defined by the active oscillation of the arteriole radius *r*. In this work, we compare spectra of the LDF signals of blood flow measured in patients and spectra of the arteriole radius oscillations calculated in the model at the different physiological conditions.

## Results

**Figure 1:**
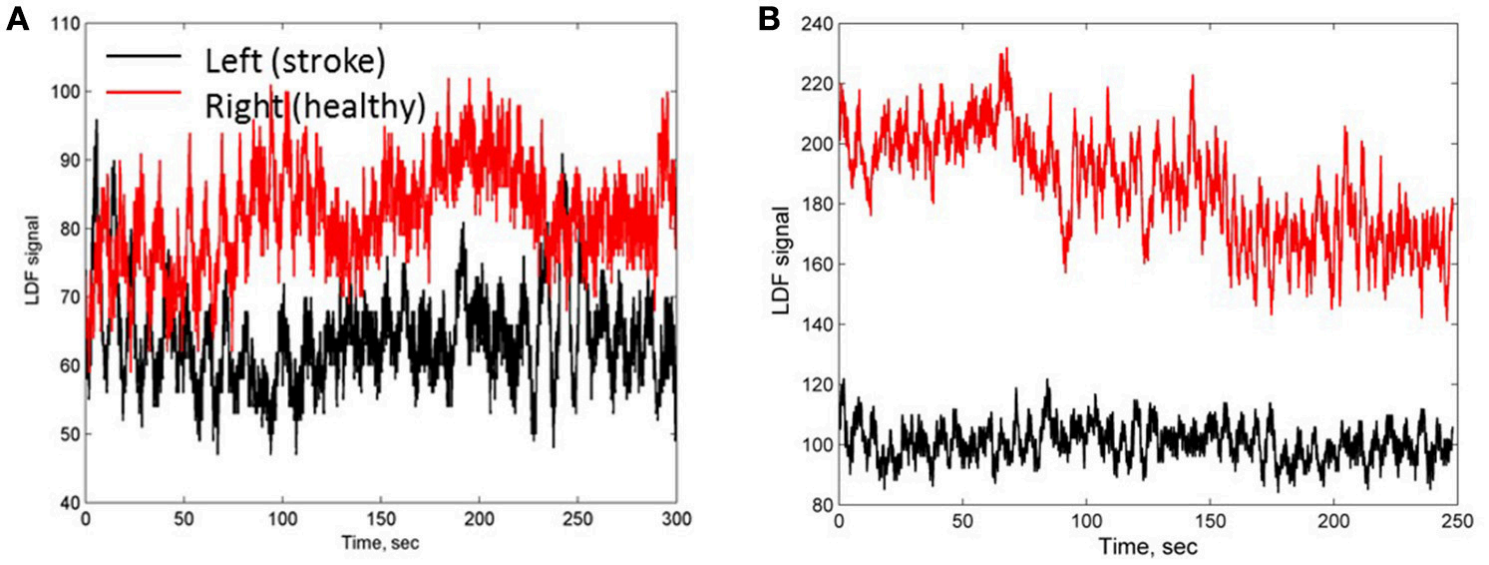
**LDF signals measured on a side of stroke affected (black line) and unaffected (red line) hemispheres of patients 1 (A)** and 2 **(B)** with a small focal AIS lesion.

LDF investigation was carried out in patients with stroke localization in the right (10 patients) and left (9 patients) cerebral hemispheres. According to LDF data, the patients after thrombolytic therapy had asymmetric haemodynamic responses measured on the right and left sides of forehead corresponding to affected and unaffected hemispheres. Two typical LDF signals obtained on both forehead sides in the ischemic patients with a small focal lesion is shown in Figures 1A,B. The LDF signals on affected and unaffected hemispheres differ between each other both in amplitude and frequency. Comparison of the LDF signals in patients with different pathology showed significantly lower perfusion value (*p* <0.05) in AIS patients (11.7 ± 5.5 pu and 13.3 ± 7.4 pu in affected and unaffected hemispheres respectively) than that in patients with chronic cerebrovascular encephalopathy (26.2 ± 4.4 pu).

**Figure 2:**
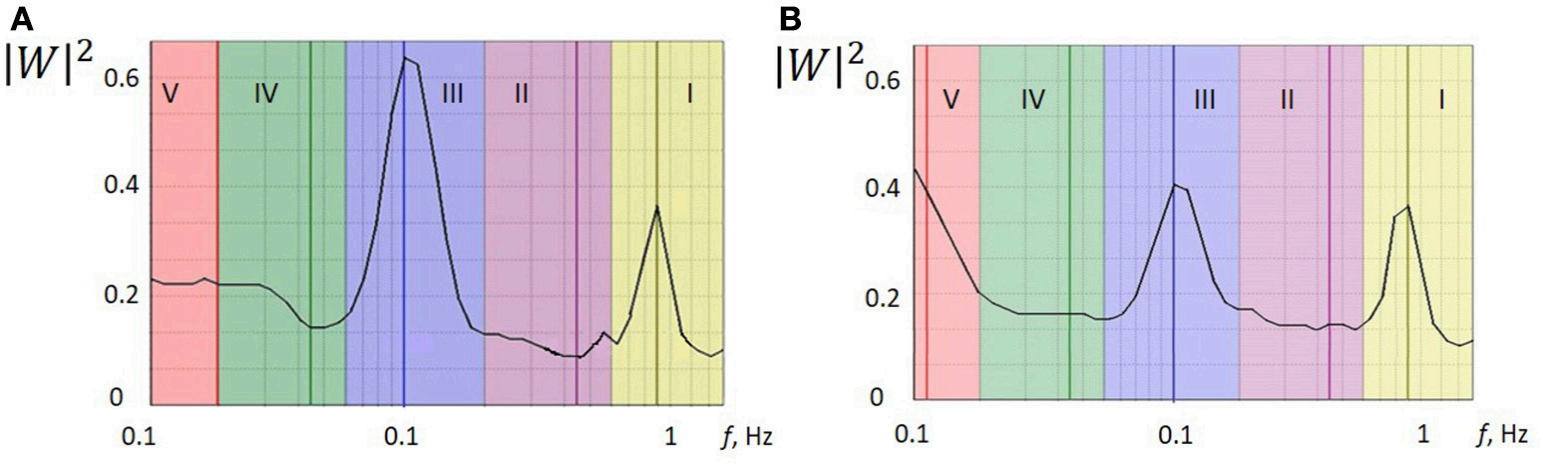
**The wavelet power spectra of the LDF signals measured on a side of stroke-affected (A)** and unaffected **(B)** hemispheres of patient 1 with a small focal AIS lesion (Figure 1A). Five physiological frequency regions are marked: (I) cardiac activity, 0.6–2 Hz; (II) respiratory activity, 0.145–0.6 Hz; (III) myogenic activity of VSMs, 0.052–0.145 Hz; (IV) microvessel innervation, 0.021–0.052 Hz; (V) endothelial activity, 0.0095–0.021 Hz.

Wavelet analysis was performed for five frequency ranges typical for LDF spectra of vasomotion: endothelial (0.0095–0.02 Hz); neurogenic (0.02–0.06 Hz); myogenic (0.06–0.16 Hz); breathing (0.16–0.4 Hz) and heart rate (0.4–1.6 Hz). The results of the wavelet analysis of the LDF signal for the patient 1 with a small focal lesion (Figure 1A) are presented in Figures 2A,B for affected and non-affected hemispheres, respectively. Amplitude and normalized amplitude of LDF wavelet spectrum corresponding to the heart rate and myogenic frequency bands were *A*_*p*_ = 0.36 and *A*_*m*_/σ = 0.57 for affected hemisphere and *A*_*p*_ = 0.36 and *A*_*m*_/σ = 0.44 for unaffected one respectively, were σ is the standard deviation of the amplitude of LDF spectrum in this region. To estimate differences in the spectral characteristic of the wavelet spectra for affected and unaffected hemispheres, we calculated the means and spreading of the normalized wavelet spectral components (A/σ) and showed this comparison in Figure S1 in Supplement.

LDF wavelet spectra measured on a side of affected hemisphere in the AIS patients after thrombotic therapy showed that the heart rate rhythm has dominated among the passive oscillations in the spectrum that indicates blood inflow to microvessel bed. Analysing the breathing rhythm frequency spectrum, we did not observe significantly high amplitude values that might occur at venular stagnation of blood and a decrease in perfusion pressure. The amplitude of the breathing rhythm did not exceed the amplitude of the heart rate rhythm of 83.3% of patients. Typical feature of the LDF wavelet spectra observed in 83.3% patients was a considerable contribution of myogenic oscillation in the frequency range of active oscillations. This can indicates the myogenic component in autoregulation of the cerebral haemocirculation and suggests pre-capillary activity and an increasing number of open capillaries (Krupatkin, 2005). Thus, enhancement of myogenic activity of arteriole smooth muscles and boost of nutritive blood flow occurred after thrombolytic therapy (Krupatkin, 2005). We have to note, that this feature of LDF wavelet spectrum was observed in only 28.5% of the patients with ischemic stroke who did not receive thrombolytic therapy.

The analysis of the LDF signals measured on a side of affected hemisphere in patients with small (<5,000 mm^3^, group 1, *n* = 5) and with large (>5,000 mm^3^, group 2, *n* = 14) focal brain lesion mapped by CT was carried out. No significant difference in the perfusion values between groups 1 and 2 was observed, while distinct difference in wavelet spectral characteristics i.e., myogenic rhythm was pronounced for patients of the group 1. This clearly indicates activation of myogenic type of the autoregulation in the resistive arterioles and the nutritive mode of microcirculation (Krupatkin, 2005). The other active rhythms in the LDF wavelet spectrum were not well distinguishable. Amplitude ratio of heart rate to breathing rhythm was *A*_*p*_/*A*_*b*_ >1. For patients of the group 2, the other active oscillatory rhythms apart from myogenic oscillations were found, namely neurogenic and rare endothelial ones, that suggests shunt component of circulation and less pronounced nutritive mode of the cerebral blood microcirculation appeared to be active (Krupatkin, 2005). In some cases, myogenic oscillations in the spectrum were not dominated. In the passive oscillation range of the spectrum, the ration of the amplitudes was *A*_*p*_/*A*_*b*_ <1 that testifies to venular stagnation (Krupatkin, 2009).

**Figure 3:**
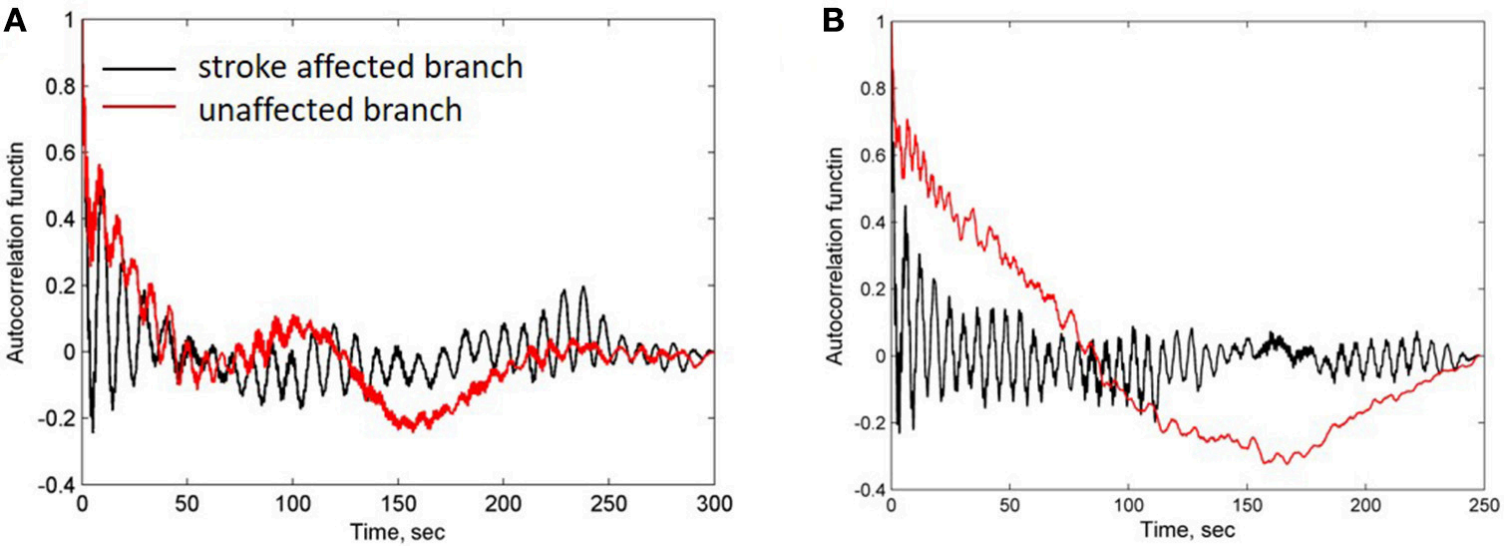
**Auto-correlation functions of LDF signals measured in patients 1 (A)** and 2 **(B)** shown in Figures 1A,B respectively. Black and red lines correspond to the stroke-affected and unaffected hemispheres, respectively.

**Figure 4:**
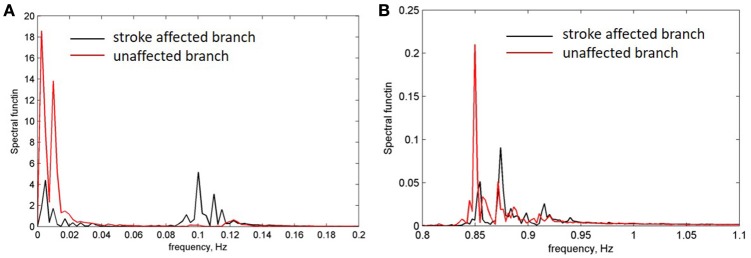
**Power density spectrum of LDF signals measured on a side of affected (black line) and unaffected (red line) branches. (A)** Frequency range of low-frequency endothelia components (0.01 Hz) and high-frequency myogenic components 0.05–0.1 Hz. **(B)** Frequency range of heart pulse (0.8–1 Hz).

For further evaluation of the spectral characteristics of the LDF signals we analyzed spectral density of the auto-correlation function of the LDF signals [Equation (3) in Methods]. Auto-correlation functions *R*(τ) of the LDF signals measured on unaffected (red line in Figures 3A,B) and stroke-affected (black line in Figures 3A,B) branches of the supraorbital arteries in two patients (Figures 1A,B) are given in Figures 3A,B. As seen, the auto-correlation functions of the LDF signals corresponding to stroke-affected and unaffected hemispheres differ from one another in their shapes and these differences are similar for both ischemic patients with a small focal lesion. Power spectral density of the auto-correlation function of LDF signals *P*(*f*_*k*_) [Equation (5)] in the range of 0.01–0.1 Hz and in the heart rate range of 1 Hz are shown in Figures 4A,B respectively for the first patient (corresponding LDF signals and auto-correlation functions shown in Figures 1A, 3A). Both auto-correlation function *R*(τ) and its power spectral density *P*(*f*_*k*_) calculated for unaffected (red lines) and stroke-affected (black lines) hemispheres significantly differ between each other (Figures 3, 4). Comparison of these results with that of the LDF wavelet analysis showed similar differences of the LDF spectra measured on unaffected and AIS-affected hemispheres.

**Figure 5:**
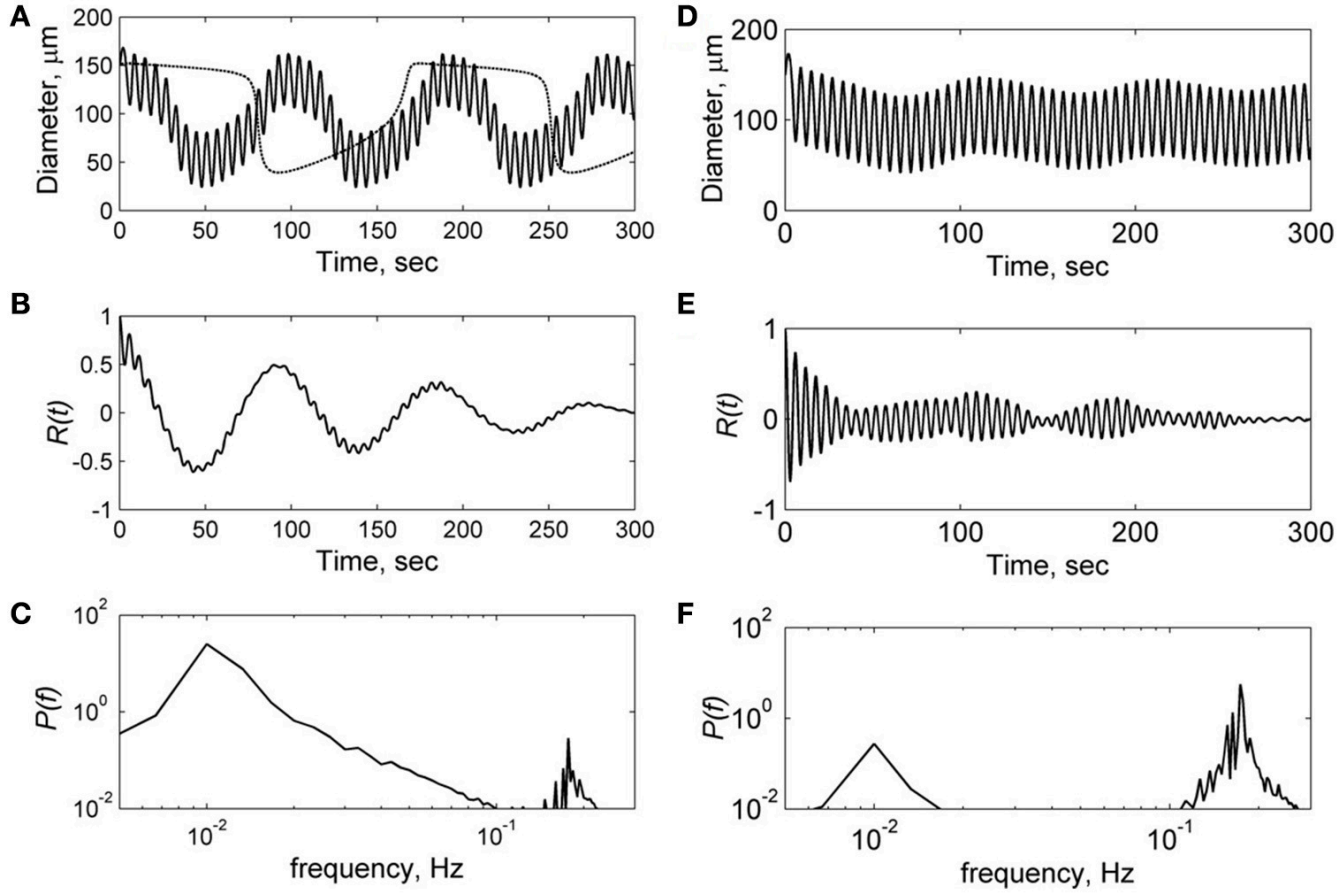
**Results of the computational modeling of microvessel diameter oscillation at low (A–C)** and high **(D–F)** amplitudes of the myogenic forced oscillation *A*_*m*_:1 kdyn/cm and 1.8 kdyn/cm respectively. **(A,D)**—diameter oscillation, **(B,E)**—auto-correlation functions *R*(*t*), **(C,F)**—power spectral density of auto-correlation functions *P*(*f*). Dot line in (A) is a solution at *A*_*m*_ = 0 corresponding to spontaneous oscillation in the unperturbed model.

To analyse the mechanism underlying the observed asymmetry in the LDF spectra in the left and right hemispheres in patients with mono-hemispheric stroke, we applied a nonlinear model of spontaneous (vasomotion) oscillation of microvessels which was developed by Arciero and Secomb (Arciero and Secomb, 2012; see Methods). This phenomenological model describes spontaneous oscillation of the arteriole diameter and blood flow under the wall tension and wall share stresses (Arciero and Secomb, 2012). In this work, authors defined a set of model parameters at which spontaneous (limit-cycle) oscillation of vascular diameter takes place and carried out bifurcation analysis of this oscillatory system (Table 3). Taking the same set of the model parameters (Table 3), we obtained spontaneous oscillations of vascular diameter in the frequency range of 0.01 Hz in this model (see dot line in Figure 5A).

Application of this model to the description of arteriole diameter oscillations in the presence of myogenic oscillations showed that the character of vasomotions significantly depends on the amplitude of myogenic oscillations. Figure 5 shows results of the calculation of arteriole diameter oscillation, auto-correlation function and its power spectrum. Calculations were carried out at low amplitude *A*_*m*_ = 1 kdyn/cm (Figures 5A–C) and high amplitude *A*_*m*_ = 1.8 kdyn/cm (Figures 5D–F) of the myogenic oscillations. At low amplitude *A*_*m*_, the diameter oscillations were obtained to be quasiperiodic, i.e., high frequency oscillations were modulated by low frequency spontaneous oscillations of 0.01 Hz (Figure 5A). At high amplitude of myogenic oscillations *A*_*m*_, high frequency oscillations prevail in the spectrum with less pronounced modulation by the low frequency of 0.02 Hz (Figure 5D). At higher amplitudes *A*_*m*_ more than 2 kdyn/cm, the modulation by high frequency disappeared because of locked modes in the forced relaxation oscillation (data not shown).

Comparison of the spectrum obtained for low and high amplitudes of myogenic oscillation revealed that amplitude of low frequency limit-cycle oscillations (0.01 Hz) decreased when the amplitude of myogenic oscillation increased (see Figure 5). These changes in spectral density is in a satisfactory agreement with experimental data on asymmetry obtained for LDF spectra, i.e., a reduction of the myogenic rhythm component in the frequency range of 0.1 Hz is accompanied by a rise of the endothelia rhythm component in the region of 0.01 Hz in the stroke-affected hemisphere compared with the unaffected one (see Figure 4). Comparison of the experimental and theoretical auto-correlation functions showed that the model is capable to satisfactorily describe the change in their shapes observed at the increasing myogenic rhythm component that occurred in the stroke-affected hemisphere (Figures 3, 5B,E).

**Figure 6:**
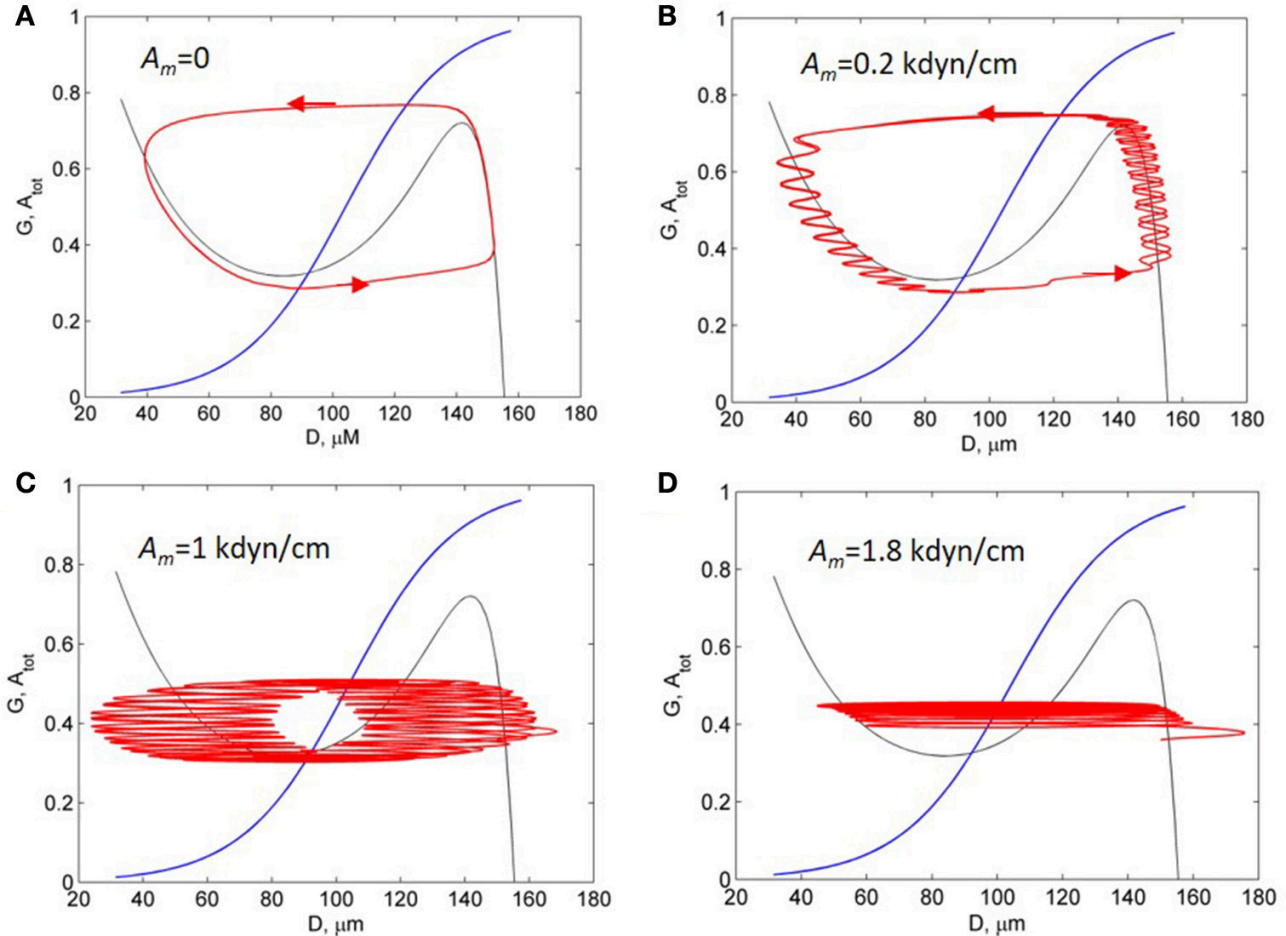
**Activation-diameter phase portrait of the vasomotion model [Equation (6)]**. Black and blue curves are the T- and A-nullcline. Red curves are trajectories of the solutions at the different amplitudes of the myogenic forced oscillation, *A*_*m*_: 0 **(A)**; 0.2 kdyn/cm **(B)**; 1 kdyn/cm **(C)**; and 1.8 kdyn/cm **(D)**.

To investigate the mechanism underlying the change in the spectrum of vessel diameter oscillations, we have calculated phase portrait of the vasomotion model [Equation (6)] and presented it at a diameter-activation (*D*/*A*) plane along with the oscillatory trajectory of the solution (Figure 6). In the absence of the myogenic oscillations, the trajectory of the solution corresponds to the limit-cycle oscillations (Figure 6A). These oscillations of vessel diameter at low frequency (0.01 Hz) has a specific saw-shape character that corresponds to the 2D limit-cycle at the phase portrait (see the corresponding solution in Figure 5A, dotted line). At low amplitude of the myogenic oscillations the limit-cycle oscillations of the vessel diameter occur at low frequency of 0.01 Hz with superimposed high-frequency oscillations of 0.1 Hz (Figure 6B). At an increased amplitude of the SMC oscillations, the blowout of the 2D oscillatory trajectory from the stable branch of D-nullcline occurs (Figures 6C,D). At a further increase in amplitude of the SMC oscillation, the 2D trajectory of the limit-cycle oscillations degenerates into a semi-one dimensional trajectory that represents high-frequency oscillations slightly modulated by low-frequency oscillations (Figure 6D).

Summarising these results, we concluded that the changes in oscillation spectrum obtained in the model results from interaction of high-frequency myogenic oscillations and low-frequency limit-cycle oscillations which are controlled by vascular tone. Increasing amplitude of myogenic oscillation causes “distortion” of a limit-cycle, weakening of low-frequency components in oscillation and prevalence of high-frequency myogenic oscillation in the vasomotion spectrum. This leads to subharmonic entrainment and locked mode in the forced relaxation oscillators. These phenomena were comprehensively investigated for the forced van der Pol relaxation oscillator (Jordan and Smith, 2007).

## Discussion

LDF investigation of patients with AIS was performed from 3 to 7 days after the onset of ischemic stroke on forehead in the supraorbital region, which supplies blood to the supraorbital artery from internal carotid artery. Analysis of the LDF signals showed that the blood perfusion in the microvasculature in the projection of the affected vascular field in AIS patients was lower compared to patients with cerebrovascular insufficiency. Wavelet analysis of LDF signals of the AIS patients revealed the blood flow increase in microcirculatory and a raise in myogenic activity of muscle-containing arterioles that predominantly indicate nutritive regime of the microcirculation after systemic thrombolytic therapy. In case of significant stroke size, the reduction of the myogenic activity and nutritive pattern of the microhaemodynamics were observed, and in some cases non-nutritive pattern and/or venular stasis were detected.

Results of the wavelet analysis of LDF signals also showed asymmetry in the LDF spectra detected in stroke-affected and unaffected hemispheres. A mechanism underlying this asymmetry was analyzed by the theoretical model of microvessel wall oscillations (vasomotions) developed in a series of publications (Ursino et al., 1996; Koenigsberger et al., 2008; Arciero and Secomb, 2012). Here we have expanded the model suggested by Arciero and Secomb (Arciero and Secomb, 2012) and applied it to describe interaction between myogenic oscillation, which as it was suggested to be induced by synchronous calcium oscillations in SMCs, and spontaneous oscillations coming from coupling regulation between endothelia cells (ECs) and SMCs. In our model, myogenic oscillations were phenomenologically considered as forcing tension applied to the vessel wall from VSMs. The model phenomenologically takes into account the modulation of SMC activity by vascular endothelium signaling (Koenigsberger et al., 2005; Delgado et al., 2010; Rapoport, 2014). One of the molecular mechanisms of this modulation may be explained through the release of vasoactive substances such as e.g., nitric oxide and/or Endotheli-1 expression as a result of activation of mechanosensitive channels in response to the shear stress (Vallance and Hingorani, 1999). Another mechanism of ECs and SMCs communication can be implemented here through calcium fluctuation in gap junction between SMCs and ECs. This mechanism was considered in the vasomotion model developed by Koenigsberger et al. (2005). The vasomotion model used in our paper [Equations (6) and (7)] phenomenologically describes ECs and SMCs communication through the negative feedback control in this coupled systems that is represented by negative terms in the right hand sides of these equations.

The coupled model of vasomotion which describes forced relaxation oscillation of active arterioles was solved numerically and spectral characteristics of its oscillatory solutions were calculated. The analysis of the power density spectrum of arteriole wall oscillations revealed two main frequency bands. The first one is in the frequency region of 0.01 Hz and corresponds to the relaxation oscillation in the coupled system. The second band in the range of 0.1 Hz is defined by forced myogenic oscillation. Analysis of the model showed that frequency patterns of vasomotion spectrum significantly depend on the amplitude of the myogenic oscillation. Amplitude of low-frequency limit-cycle oscillation in the range of 0.01 Hz decreases when amplitude of myogenic oscillations increases. This transformation of the spectrum at low- and high-frequency regions is defined by the phenomenon of subharmonic entrainment and locked mode in the forced relaxation oscillatory models, that was established well for coupled nonlinear models such as the forced van der Pol model (Jordan and Smith, 2007).

Note, in the model, we took into account only interaction between myogenic and endothelial oscillations and did not consider their coupling with neurogenic modes of vasomotions. The theoretical approach to consideration of this coupling in vasomotion models was discussed in Stefanovska (1999). Authors suggested that the coupling of the different modes ensures a coordinative control of the vascular function by different physiological mechanisms. Authors also mentioned that a study of these mode coupling can bear practical implications for diagnosis of microvascular circulation abnormalities (Stefanovska, 1999).

The transformation of the spectral density of vasomotion obtained in the model at the change of myogenic activity are in a satisfactory agreement with experimental data on the the LDF spectra measured in the stroke affected and unaffected hemispheres in AIS patients. A decrease in myogenic component in the frequency range of 0.1 Hz is accompanied by an increase in endothelia component in the region of 0.01 Hz of the spectra. Base on this comparison, we assumed that declining in myogenic oscillation amplitude observed in the hemisphere affected by AIS is accompanied by an increasing amplitude of low frequency component in the frequency range of 0.01 Hz. Note that similar reciprocal relationship among different components of active oscillations in the LDF wavelet spectra was observed in LDF investigation of microcirculation (Krupatkin, 2009). Author assumed that disappearance of the myogenic components in the LDF wavelet spectrum is accompanied by increase in other components of the spectrum. The application of the vasomotion model (Arciero and Secomb, 2012) to analyse experimental data allowed us to suggest the possible mechanism of intra-spectral changes of the LDF spectra measured in the patients with unilateral ischemic stroke.

In our theoretical description of the clinical data, we assumed identical parameters of the cerebrovascular systems of both hemispheres in healthy conditions. In the model, the asymmetry in the LDF signal arises from a relative difference in myogenic regulation of cerebrovascular dynamics in different hemispheres. Imbalance in endothelial signaling between hemispheres was not taken into account in the model. Although this factor related to endothelia dysfunction may affect asymmetry of the LDF signals in the endothelia frequency range (VI) of 0.005–0.0095 Hz. Another restriction of our theoretical approach to modeling of the LDF data is that we did not consider network vessel structure which forms the resultant LDF signal. In the simplified approach, we used the model of a single vessel with fixed pressure. To expand this model to the response of network vessel structure, multi-compartment approaches can be used which were developed to model vascular pathway (Ursino et al., 1996; Lanzarone et al., 2007; Arciero and Secomb, 2012).

A limitation of our experimental investigation is the absence of a control group for reliable estimation of inter-hemispheric variability (IHV) for AIS patients. This problem was also arisen in previous studies of the IHV and CA in LFO range of vasomotions (Phillip et al., 2012, 2013). In order to solve this problem, investigation of the IHV in healthy cohort as a control group was carried out using NIRS method (Phillip et al., 2012, 2013). Based on the absence of noticeable inter-hemispheric difference in the obtained data for the healthy cohort, authors suggested full synchronization of cerebrovascular regulation between hemispheres for healthy population and referred the observed IHV to impairment of perfusion and CA in affected and contralateral hemispheres in patients with occlusive disease (Phillip et al., 2013). Our data on the IHV in AIS patients conform in part with the IHV observation in LFO frequency region of vasomotion in the investigation of patients with AIS, carotid artery stenosis and cerebrovascular occlusion disease (COD) performed by NIRS and transcranial Doppler sonography (Reinhard et al., 2003; Phillip et al., 2013; Phillip and Schytz, 2014). In these studies, abrogation in inter-hemispheric synchronicity was defined mainly by the measurement of phase shift between ABP and oxyHb (Phillip et al., 2012; Phillip and Schytz, 2014) and between ABP and CBF (Reinhard et al., 2003) in the LFO range that showed impairment of CA at these cerebrovascular diseases. Amplitude ratio of ABP to oxyHb in the LFO range of spectrum was measured in COD (Phillip et al., 2012) and AIS patients (Phillip and Schytz, 2014) that showed higher ratio for the affected side compared to the contralateral one (ratio 1.3 ± 0.3 for AIS). While the measurement of the inter-hemispheric amplitude of oxyHb in the LFO range in AIS patients showed no significant difference in amplitude of the oxyHb LFOs on the stroke-affected side compared to the contralateral side (0.014 ± 0.03 vs. 0.017 ± 0.03; Phillip and Schytz, 2014). This result contradicts our data on declining spectral components in the frequency region near 0.1 Hz measured in stroke-affected hemisphere in 83.3% ischemic patients. Authors explained the absence of the amplitude difference in the stroke patients possible compensatory dilatation of arterioles distal to ischemic area as well as by LFOs decreasing with age and macroangiopathy in patient group (Phillip and Schytz, 2014). This discrepancy between our results and the results obtained in Phillip and Schytz (2014) requires further investigation of the inter-hemispheric variation with a wide range of patients by spectral characteristic monitoring in pre and in-treatment period up to complete or partial re-establishing of inter-hemispheric synchronization due to compensatory processes in affected and/or non-affected hemispheres.

## Conclusion

The preliminary results obtained in this pilot study showed that the inter-hemispheric variation in intra-spectral characteristics of LDF signals measured in AIS patients can be a prognostic factor for diagnostics of the cerebrovascular disease. Wavelet analysis of the LDF signals and theoretical investigation of vasomotions revealed that such prognostic factor may be the relationship between the spectrum components lying in the physiological relevant frequency bands related to myogenic and endothelial oscillations. Exploitation of the intra-spectral correlations in the inter-hemispheric variability of the LDF spectra can give useful information additionally to inter-spectral characteristics such as phase shift and correlation coefficients obtained in NIRS and LDF investigation of cerebrovascular autoregulation impairments in patients with cerebrovascular diseases. For further development of this technique and translation of it to clinics, we suggest performing long term monitoring of the inter-hemispheric variation by LDF and NIRS and verify the obtained data by another group of patients with double blinded clinical trial. Spectral data collected in this monitoring will be valuable for estimation of population variants in the inter-hemispheric variability and reconciliation of the results obtained in different clinical trials. Analysis of these data along with computational modeling of vasomotion will help to understand physiological mechanism underlying inter-hemispheric variation in spectral characteristics at pre- and in-treatment period. The results of this analysis will facilitate design predictive and preventive intra- and inter-spectral markers of the prevention, progression and treatment of cerebrovascular diseases.

## Author contributions

ER, VS, AK, and SS conceived main idea of the study; VS and AK designed the experiments; AA and MZ carried out a clinical investigation and data acquisition; AG conducted data analysis, modeling, and drafted the manuscript; SS, VS, and AK analyzed, interpreted the data, and contributed to the preparation of a final version of the manuscript.

### Conflict of interest statement

The authors declare that the research was conducted in the absence of any commercial or financial relationships that could be construed as a potential conflict of interest.
