# Supplementary material for: Bifurcation in Blood Oscillatory Rhythms for Patients with Ischemic Stroke: A Small Scale Clinical Trial using Laser Doppler Flowmetry and Computational Modeling of Vasomotion
Source: Front Physiol. 2017 Mar 23;8:160. doi: 10.3389/fphys.2017.00160 (PMC5362641; doi:10.3389/fphys.2017.00160)
Supplement: Supplementary file 1 [file Presentation1.PDF]

Supplement

Bifurcation in blood oscillatory rhythms for patients with ischemic stroke: a small scale clinical trial using laser Doppler flowmetry and computational modelling of vasomotion

A. Goltsov, A.V. Anisimova, M. Zakharkina, A.I. Krupatkin, V.V. Sidorov, S.G. Sokolovski, E.U. Rafailov

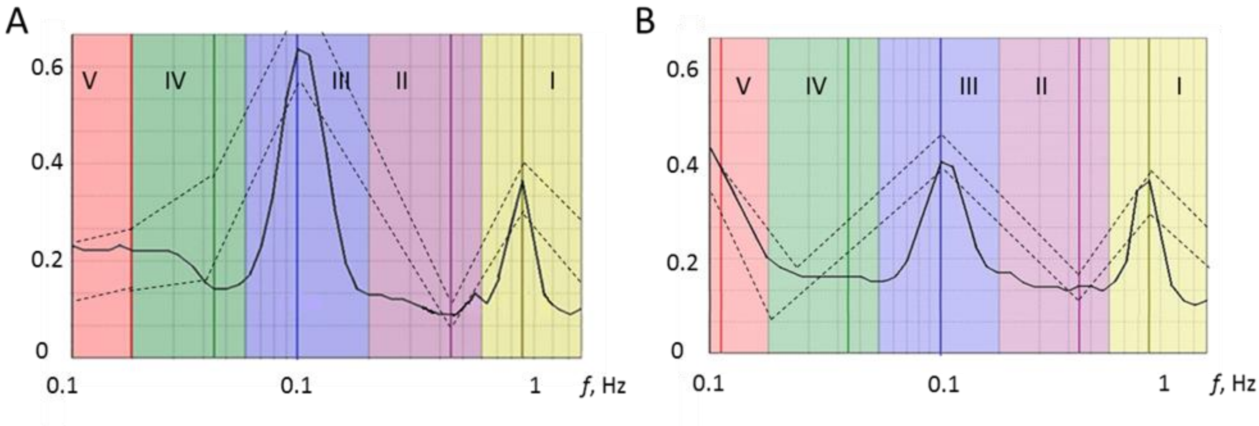

Fig. S1. The wavelet power spectra of the LDF signals measured on a side of stroke-affected (A) and unaffected (B) hemispheres of patient 1 with a small focal AIS lesion (Fig. 1A). Dashed lines show spreading of the LDF wavelet spectra for normalized amplitudes of the heart rate and myogenic picks, A/σ, where σ is the standard deviation of the amplitudes A. The upper and lower dashed lines correspond to upper and lower boundaries of the normalized spectra.
